# AhR-activating pesticides increase the bovine ABCG2 efflux activity in MDCKII-bABCG2 cells

**DOI:** 10.1371/journal.pone.0237163

**Published:** 2020-08-07

**Authors:** Lydia Kuhnert, Mery Giantin, Mauro Dacasto, Sandra Halwachs, Walther Honscha

**Affiliations:** 1 Institute of Pharmacology, Pharmacy and Toxicology, Faculty of Veterinary Medicine, University Leipzig, Leipzig, Germany; 2 Division of Veterinary Pharmacology and Toxicology, Department of Comparative Biomedicine and Food Science, University of Padua, Agripolis Legnaro (Padua), Italy; Wayne State University, UNITED STATES

## Abstract

In bovine mammary glands, the ABCG2 transporter actively secretes xenobiotics into dairy milk. This can have significant implications when cattle are exposed to pesticide residues in feed. Recent studies indicate that the fungicide prochloraz activates the aryl hydrocarbon receptor (AhR) pathway, increasing bovine ABCG2 (bABCG2) gene expression and efflux activity. This could enhance the accumulation of bABCG2 substrates in dairy milk, impacting pesticide risk assessment. We therefore investigated whether 13 commonly used pesticides in Europe are inducers of AhR and bABCG2 activity. MDCKII cells expressing mammary bABCG2 were incubated with pesticides for up to 72 h. To reflect an *in vivo* situation, applied pesticide concentrations corresponded to the maximum residue levels (MRLs) permitted in bovine fat or muscle. AhR activation was ascertained through CYP1A mRNA expression and enzyme activity, measured by qPCR and 7-ethoxyresorufin-Ο-deethylase (EROD) assay, respectively. Pesticide-mediated increase of bABCG2 efflux activity was assessed using the Hoechst 33342 accumulation assay. For all assays, the known AhR-activating pesticide prochloraz served as a positive control, while the non-activating tolclofos-methyl provided the negative control. At 10-fold MRL concentrations, chlorpyrifos-methyl, diflufenican, ioxynil, rimsulfuron, and tebuconazole significantly increased CYP1A1 mRNA levels, CYP1A activity, and bABCG2 efflux activity compared to the vehicle control. In contrast, dimethoate, dimethomorph, glyphosate, iprodione, methiocarb and thiacloprid had no impact on AhR-mediated CYP1A1 mRNA levels, CYP1A activity or bABCG2 efflux. In conclusion, the MDCKII-bABCG2 cell model proved an appropriate tool for identifying AhR- and bABCG2-inducing pesticides. This provides an *in vitro* approach that could reduce the number of animals required in pesticide approval studies.

## Introduction

In Germany, about 32 000 t of active pesticide substances are used in conventional agriculture each year [1]. Food producing animals are exposed to these pesticides through the ingestion of residues left on plants [2–5]. Acute intoxications are rare given low concentrations of residues [3], but long-term exposure could disturb cellular pathways leading to chronic diseases such as cancer and neurodegenerative disorders [6]. To protect the consumer, animal models are used to assess the potential risk of pesticide residues [7].

Several pesticides have been shown to impact transcription factors, such as the aryl hydrocarbon receptor (AhR) [8,9]. AhR belongs to the bHLH/PAS family of transcription factors and is localized to the cytoplasm as an inactive heterodimer of HSP90 (heat shock protein 90), XAP2 (hepatitis B virus protein X-associated protein2), and p23 [10]. After ligand-binding, the chaperones are released and the ligand-AhR-complex translocates to the nucleus. The complex dimerizes with its required partner ARNT (aryl hydrocarbon nuclear translocator) and binds to special motifs (DRE, dioxin response elements) in the 5'-untranslated region (UTR) resulting in increased AhR target gene expression [10,11]. The AhR target gene cytochrome P450 1A1 (CYP1A1) is currently used as a biomarker to identify AhR-activating substances [12–15]. AhR activation increases detoxification to protect the organism from xenobiotic exposure by influencing the gene expression and activity of efflux transporters, such as the ABCG2 transporter (ATP-binding cassette subfamily G 2, Breast Cancer Resistance Protein, BCRP) [11,16–18].

In bovine mammary glands, the bovine ABCG2 (bABCG2) transporter is localized in the apical membrane of alveolar epithelial cells [19] representing the main route of xenobiotics into milk [20]. The substrate spectrum contains commonly used veterinary drugs, such as antibiotics, anthelmintics [21–26], and other toxic compounds including mycotoxins [27]. Halwachs et al. (2013) showed that TCDD (2,3,7,8-tetrachlorodibenzo-*p*-dioxin) and the fungicide prochloraz increased bABCG2 transport activity in primary bovine mammary epithelial cells (PBMEC). Specific AhR DREs were identified in the 5`-UTR of the bABCG2 gene. TCDD and prochloraz activated these DREs, increasing both expression and activity of the AhR target gene CYP1A. It was therefore concluded that AhR mediated the induction of bABCG2 activity [28]. Another study with Madin-Darby canine kidney II cells overexpressing bABCG2 (MDCKII-bABCG2) also demonstrated a TCDD- and prochloraz-mediated activation of AhR induced bABCG2 mRNA expression and efflux activity [29]. Therefore, the use of these pesticides could lead to an accumulation of potentially harmful ABCG2 substrates in dairy milk, elevating consumer risk.

While human drug approval processes investigate drug-drug interactions, this is not part of the pesticide approval process [30,31]. The first step investigates pesticide-mediated induction of efflux transporters through i*n vitro* assays [31,32]. However, very few studies have been published on pesticide-mediated regulation of drug transporters [32], and no species-specific *in vitro* models are available. Instead, pesticide assessment is more commonly based on animal experiments with lactating goats or cows. Nevertheless, replacement, reduction and refinement of animal studies (the 3R principle) is highly encouraged. This study proposes an *in vitro* MDCKII-bABCG2 cell model for identifying AhR-activating and bABCG2-inducing pesticides, implementing the 3R principle in practice. The study subsequently used the cell model to asses thirteen pesticides approved for use in the European Union and commonly found in conventional agriculture, particularly Germany.

## Material and methods

### Chemicals and solvents

Milli-Q water was prepared using a Millipore Synergy UV water purification system (Millipore S.A.S., Molsheim, France). Solvents at analytical grade were purchased from Carl Roth (Karlsruhe, Germany) and Merck Millipore (Darmstadt, Germany). All other chemicals were obtained from Sigma-Aldrich (Darmstadt, Germany), except for methiocarb (Dr. Ehrenstorfer, Augsburg, Germany) and 2,3,7,8-tetrachlorodibenzo-*p*-dioxin (TCDD, AccuStandard, New Haven, USA). Stock solutions of pesticides, TCDD, and 2,2’,4,5,5’-pentachlorobiphenyl (PCB101) were prepared with declared solvents (Table 1) in accordance with their maximal solubility listed in Pesticide Properties data base [33] or PubChem [34]. The prepared pesticide concentrations were subsequently diluted with cell culture medium to the same percentage level as the solvents stated in Table 1.

**Table 1:** Selected compounds, 1-fold MRL (maximum residue level) concentrations, and solvents used for their dissolution.

	Substance group	Lot no.	Purity	MRL muscle^a^/fat^b^		Solvent		Reference
				[mg/kg]	[nM]		[%]	
**TCDD**	Dioxin	215031440		n. a.	1	toluene	0.1	[28]
**PCB101**	Polychlorinated biphenyl	SZBC032XV	98.0	n. a.	10	DMSO	0.1	[28]
**Prochloraz**	Imidazole	SZBA112X	99.1	0.03^b^	80	ethanol	0.1	[35]
**Tolclofos-methyl**	Thiophosphoric ester	SZBA323XV	97.9	0.01^a,b^	33	methanol	0.1	[36]
**Chlorpyrifos-methyl**	Organophosphate	SZBC109XV	99.9	0.01^a,b^	37	methanol	0.1	[37]
**Diflufenican**	Carboxamide	SZBC048XV	97.9	0.01^a,b^	25	methanol	0.2	[38]
**Dimethoate**	Organophosphate	SZBC243XV	99.5	0.01^a,b^	44	methanol	0.2	[39]
**Dimethomorph**	Morpholine	SZB9069XV	99.0	0.02^a,b^	52	methanol	0.2	[40]
**Glyphosate**	Phosphonate	SZBC164XV	99.9	0.05^a,b^	296	milli-Q water	0.4	[36]
**Ioxynil**	Hydroxybenzonitrile	SZB8114XV	99.8	1.00^a,b^	2,700	methanol	0.5	[36]
**Iprodione**	Dicarboximide	SZBC174XV	99.5	0.05^a,b^	100	methanol	0.1	[41]
**Methiocarb**	Carbamate	10630	99.5	0.01^a,b^	44	xylene	0.1	[42]
**Rimsulfuron**	Sulfonylurea	SZBC047XV	99.9	0.05^a,b^	116	ethyl acetate	0.1	[43]
**Tebuconazole**	Triazole	SZBB055XV	99.5	0.10^a,b^	325	toluene	0.1	[36]
**Thiacloprid**	Neonicotinoid	SZBC180XV	99.9	0.05^a,b^	198	ethyl acetate	0.1	[36]

n.a. not available, COM (European Commission), DMSO (dimethyl sulfoxide), EFSA (European Food Safety Authority)

### Selection of pesticides and their concentrations

Commonly-used pesticides were selected for testing based on three key criteria: (1) the substance is approved for use in the European Union [44,45], (2) the substance is present in the “top ten list” of pesticides [46], and (3) the substance has been identified in national monitoring programs as regularly exceeding maximum residue levels (MRLs) in food [47–50]. During the study the European approval of ioxynil and iprodione expired in 2015 and 2018, respectively [45]. In line with previous studies [8,9,28,29,51], the known AhR-activating pesticide prochloraz served as a positive control, while the non-activating tolclofos-methyl provided the negative control. To reflect the *in vivo* situation, applied pesticide concentrations corresponded to the MRLs permitted in bovine fat or muscle (Table 1).

### Cell culture

All studies were performed with Madin-Darby canine kidney II cells (MDCKII) that overexpressed the bovine ABCG2 efflux transporter, as developed by Wassermann et al. 2013 [22]. Cells were cultivated in MEM medium with Earle’s Salts (2.2 g/L NaHCO_3,_ stable glutamine; Biochrom, Berlin, Germany), supplemented with 10% (*v/v*) fetal calf serum (Life Technology, Karlsruhe, Germany), 1% (*v/v*) non-essential amino acids (Biochrom, Berlin, Germany), 100 U/mL penicillin, 100 μg/mL streptomycin (Biochrom, Berlin, Germany), and grown at 37°C and 5% CO_2_. Cells were sub-cultured using 0.05% trypsin/0.02% EDTA (Biochrom, Berlin, Germany) every 3 to 4 days, up to a total of 14 passages.

### Cytotoxicity (WST-1 assay)

Cytotoxicity of selected pesticides was determined by water-soluble tetrazolium 1 (WST-1) cytotoxicity assay (Roche, Mannheim, Germany). MDCKII-bABCG2 cells (3 x 10^4^ cells/mL) were seeded in 96-well plates (TPP, Trasadingen, Switzerland). After 24 h, cells were incubated with increasing concentrations (up to 500-fold MRL) of the selected pesticides for 72 h. Cells treated with 0.1% Triton X-100 served as positive control and untreated MDCKII-bABCG2 cells were used as negative control. The WST-1 assay was performed as described by Halwachs et al. (2013) [28]. SigmaPlot 11.0 (Systac Software, San Jose, CA, USA) was used to calculate the IC_50_ (inhibitory concentration), defined as the pesticide concentration that reduces cell viability to 50%. Data are presented as mean ± SEM, calculated from at least two independent experiments (N ≥ 2) with over 12 technical replicates per experiment (n ≥ 12).

### Gene expression analysis (qPCR)

For gene expression analysis, 5 x 10^4^ MDCKII-bABCG2 cells per mL were seeded in cell culture dishes (100/20 mm, Greiner Bio-one GmbH, Frickenhausen, Germany). 6–8 h after seeding, cells were treated either with pesticides in 0.1-, 1- and 10-fold MRL concentrations (Table 1) or its corresponding solvent. The treatment was renewed once a day. TCDD (1 nM, 10 nM) and PCB101 (10 nM, 100 nM) were selected as the AhR-activating and non-activating compounds, respectively. After a total of 72 h of incubation, cells were washed twice with PBS, and then lysed with 1 mL of 0.05% trypsin/0.02% EDTA. The proteolytic cell dissociation was stopped with cell culture medium. The suspension was centrifuged (2000 rpm, 5 min), the supernatant removed and the pellet washed twice with PBS. Afterwards, cell density was adjusted to 3 x 10^6^ cells, and 400 μL RNAlater^®^ solution (Invitrogen, Life Technologies, Carlsbad, USA) was added to the pellet. After an overnight storage at 4°C, pellets were transferred to -20°C for subsequent total RNA extraction.

After RNAlater^®^ removal, total RNA was isolated using the TRIzol^®^ reagent (Invitrogen, Life Technologies, Milan, Italy) coupled with the RNeasy Mini kit (Qiagen, Hilden, Germany) for the aqueous phase purification, following manufacturer’s procedures. A DNase digestion step with RNase-free DNase (Qiagen, Hilden, Germany) was also performed. The nucleic acid was evaluated in terms of quality and quantity using the Nanodrop ND1000 spectrophotometer (Nanodrop Technologies, Wilmington, DE) and 1% agarose gel electrophoresis under denaturing conditions. Afterwards, 1 μg of total RNA was reverse-transcribed using the High Capacity cDNA Reverse Transcription kit (Life Technologies, Milan, Italy) following manufacturer’s instructions. Finally, the cDNA samples were stored at -20°C until use.

To assess the effect of pesticides at the gene expression level, AhR (ENSCAFT00000003863), aryl hydrocarbon receptor repressor (AhRR, ENSCAFT00000039404), ARNT (ENSCAFT00000019492), CYP1A1 (ENSCAFT00000028474) and cytochrome P450 1B1 (CYP1B1, ENSCAFT00000009970) were chosen as candidate genes for mRNA analysis (Table 2). Mitochondrial ATP synthase 5 (ATP5B, ENSCAFT00000000224), vacuolar protein trafficking and biogenesis associated homologue (CCZ1, ENSCAFT00000024533), hypoxanthine phosphoribosyl transferase 1 (HRPT1, ENSCAFT00000002627), ribosomal protein L8 (RPL8, ENSCAFT00000002627), ribosomal protein L32 (RPL32, ENSCAFT00000046893) and ribosomal protein S5 (RPS5, ENSCAFT00000003710) were used as internal control genes (ICGs, S2 Table).

**Table 2:** Oligonucleotides and UPL probes used for gene expression analysis.

Gene	Primer sequence (5'-3')	Primer concentration (nM)	Human UPL probe	Amplicon size (bp)	Reference
AHR	F: cttcgtgtgccgactaaggt	300	#120	63	[52]
	R: tggaaattcattgccagaaa	300			
AHRR	F: attttatgcgtcagcaacaatc	300	#165	68	[52]
	R: tgcatcacatccgtctgg	300			
ARNT	F: ccacttggacccctagcac	300	#62	60	[52]
	R: cttggctgtagcctgagca	300			
CYP1A1	F: agggacgttgcgtctttgt	300	#59	65	[52]
	R: cgggttaccccatagcttct	600			
CYP1B1	F: gacgccttcatcctctcg	600	#70	82	[52]
	R: gcacgtactccatgtccaac	300			
ATP5B	F: tctgaaggagaccatcaaagg	600	#120	74	[53]
	R: agaaggcctgttctggaagat	600			
CCZ1	F: tgaagcactgcatttaattgtttat	600	#148	96	[54]
	R: cttcggcaaaaatccaatgt	600			
HPRT1	F: tgctcgagatgtgatgaagg	300	#62	192	[53]
	R: tcccctgttgactggtcatt	600			
RPL8	F: ggacggagctgttcatcg	300	#137	90	[54]
	R: gcacattgcctatgttgagc	300			
RPL32	F: ccggaagttcctagtccaca	600	#146	78	Designed ex novo
	R: gcaatctctgcacaataagacttg	600			
RPS5	F: ccggaacatcaagactattgc	300	#136	72	[54]
	R: gaattggaagagcccttgg	300			

Gene expression levels were determined using previously validated and published quantitative real time PCR (qPCR) assays [52–54] except for RPL32, for which oligonucleotides and the corresponding Universal Probe Library (UPL) probe were designed *ex novo* using the UPL Assay Design center web service (Roche, Basel, Switzerland). The oligonucleotide sequences and concentrations, amplicon sizes and UPL probes are listed in Table 2. For evaluating qPCR performance, standard calibration curves were generated by amplifying decreasing amounts of MDCKII cDNA diluted at 3-fold intervals. Standard curve analyses of target genes and ICGs showed high test linearities (error < 0.2) and acceptable amplification efficiencies (comprised in the range between 90% and 110%). The ICGs’ amplification efficiency was equal to that of the target genes. The main qPCR parameters (efficiency, linearity and dynamic range) are reported in S1 Table. The qPCR reactions were performed using LightCycler® 480 (Roche, Basel, Switzerland), clear LightCycler® 480 Multiwell Plate 96 and standard PCR conditions. Assay-dependent forward and reverse primer concentrations were mixed with 1X LightCycler® 480 Probe Master, then the selected UPL probe (200 nM final concentration) and 2.5 ng of cDNA were added to a final volume of 10 μL. Crossing point (C_P_) or cycle number at detection threshold values were acquired using the LightCycler® 480 software release 1.5.0 using the second derivative maximum method [55]. The relative quantification of obtained data was performed using the ΔΔCt method [56]. For the normalization step, only ICGs not affected by the treatment were used for each compound (S1 Table). The selection criterion was the absence of a statistically significant difference in the arithmetic mean of ICGs between control and treated cells. Data were normalized against the vehicle control which was set as 1. Relative quantification values (RQ) were expressed in arbitrary units (AU) as mean ± SEM from three independent experiments with two technical replicates per experiment (N = 3, n = 6).

### CYP1A activity (EROD assay)

The CYP1A enzyme reduces 7-ethoxyresorufin to resorufin quantifiable through spectrofluorometry [13]. The EROD assay was therefore chosen to investigate a pesticide-mediated induction of the AhR target gene CYP1A1. Cells were seeded in 24-well plates (Sarstedt, Nümbrecht, Germany) at a density of 3 x 10^4^ cells/mL and treated with pesticides, solvents, TCDD or PCB101 as stated for qPCR analysis. After incubating the cells with pesticides for 72 h EROD activity was measured according to Donato et al. (1993) [13], with minor modifications. Cells were washed three times with warm PBS buffer (Biowest SAS, Nuaillé, France), then incubated with MEM Earle’s medium, supplemented with 10 nM dexamethasone, 16 μM 7-ethoxyresorufin, and 10 μM dicoumarol for 2 h (75 rpm, 37°C, 5% CO_2_). Cell supernatants (90 μL) and resorufin reference standards were transferred to a 96-well plate (TPP, Trasadingen, Switzerland). Cell supernatant treated only with EROD-medium for 5 min was used as a background. To reduce conjugates of formed resorufin, 30 μL 0.1 M sodium acetate buffer (pH 4.5), containing 15 fishman units of β-glucuronidase and 120 roy units of arylsulfatase (Roche, Mannheim, Germany), was added for 1 h (75 rpm, 37°C, 5% CO_2_). Following incubation, 240 μL ethanol was added to each well and centrifuged for 10 min at room temperature (3000 rpm). The formed resorufin was detected by spectrofluorometry (540 nm excitation/595 nm emission wavelengths, Tecan Genios/Tecan Infinite F200 Pro, Crailsheim, Germany) and EROD activity was calculated as pmol/min per mg protein. Protein amounts were determined by bicinchoninic acid assay (BCA, Thermo Scientific, Rockford, USA) following the manufacturer’s instructions. Data were normalized against the vehicle control which was set as 1 and expressed as mean ± SEM from three independent experiments with three technical replicates per experiment (N = 3, n = 9).

### bABCG2 efflux activity (Hoechst 33342 accumulation assay)

The Hoechst 33342 accumulation assay was used to detect bABCG2 efflux activity. The assay protocol was adapted from that published by Halwachs et al. (2014) [51]. MDCKII-bABCG2 cells were seeded in 96-well plates (TPP, Trasadingen, Switzerland) at a density of 2 x 10^4^ cells/mL. After 6–8 h, cells were treated with 1-fold MRL concentration of prochloraz or 10-fold MRL concentration of tolclofos-methyl for 12 h to 48 h. A 10-fold MRL incubation with chlorpyrifos-methyl, diflufenican, ioxynil, rimsulfuron and tebuconazole was conducted for 48 h. Controls were treated with respective solvents (Table 1). Simultaneous incubations with the ABCG2 inhibitor Ko143 (5 μM) were performed to prove the involvement of the bABCG2 transporter. Subsequently, MDCKII cells were washed twice with warm PBS followed by exposure to MEM Earle´s medium supplemented with Hoechst 33342 (5 μM, Biochemica Applichem, Darmstadt, Germany), either in the presence or absence of Ko143 (5 μM) for 30 min in a shaking incubator (150 rpm, 37°C, 5% CO_2_). Cells were subsequently washed twice with cold PBS, lysed with 0.1% SDS/PBS, and the intracellular amount of fluorescent dye Hoechst 33342 was measured (360 nm excitation/465 nM emission wavelengths, Tecan Infinite F200 Pro, Crailsheim, Germany). The relative fluorescence units (RFU) per mg protein were calculated by comparing pesticide-treated cells to solvent-treated control cells. Protein amounts were quantified by BCA. Three independent experiments were carried out, each containing six technical replicates, and data were expressed as mean ± SEM (N = 3, n = 18).

### Statistical analysis

A statistical analysis of the gene expression data was performed on three independent experiments (N = 3, n = 6). The results from the control and treated cells (two different concentrations) were compared for each compound using one-way analysis of variance (ANOVA) followed by Tukey’s post-hoc test (GraphPad Prism 5 software, San Diego, California, USA). Data from the EROD (N = 3, n = 9) and Hoechst 33342 assays (N = 3, n = 18) were examined by one-way ANOVA with Fisher-LSD post-hoc test. The level of significance was set as p ≤ 0.05.

## Results and discussion

### Cytotoxicity of pesticides, TCDD and PCB101

The cytotoxicity of the selected pesticides and their solvents was determined through WST-1 assay. An IC_50_ value was only able to be calculated for ioxynil (IC_50_ ~ 97.5 μM, S1B Fig). A decreased cell viability was also detected for prochloraz- and tolclofos-methyl-treated cells in 100- and 500-fold MRL concentrations without a calculable IC_50_ value (S1A Fig). In general, no cytotoxic effects of the chosen solvents or pesticides were observed from 1- to 10-fold MRL concentrations in MDCKII-bABCG2 cells (S1C, S1D and S2A–S2C Figs). The cytotoxicity of TCDD, PCB101 and their solvents has been previously determined in MDCKII-bABCG2 cells [29].

### Validation of MDCKII-bABCG2 cells to known AhR ligands

Contaminants such as polychlorinated hydrocarbons bind to AhR and activate the AhR pathway [10,15]. Previous studies have shown that the known AhR-activating ligand TCDD induced the bABCG2 efflux activity in PBMEC and MDCKII-bABCG2 cells while the non AhR-activating compound PCB101 did not [28,29]. In the present study, MDCKII-bABCG2 cells were treated with TCDD (1 nM, 10 nM) and PCB101 (10 nM, 100 nM) for 72 h to determine AhR activation by known AhR target genes. As shown in S3 Fig, TCDD significantly increased CYP1A1 (4-fold, S3A Fig), CYP1B1 (3-fold, S3B Fig), and AhRR (2-fold, S3C Fig) mRNA levels in both applied concentrations compared to the control. AhR mRNA levels were not affected by TCDD (S3D Fig). These results are in line with existing literature which identified CYP1A1, CYP1B1, and AhRR as typical AhR target genes [10,57,58]. TCDD induced CYP1A enzyme activity has also previously been shown in MDCKII-bABCG2 cells [29].

While PCB101 did not affect the mRNA expression of CYP1B1 or AhRR, CYP1A1 was significantly down-regulated (S4 Fig). However, in previous EROD studies, no alteration of CYP1A enzyme activity was detected in MDCKII-bABCG2 cells [29]. Post-transcriptional regulation by dynamic RNA modification may explain why the down-regulation of CYP1A1 mRNA by PCB101 does not decrease CYP1A enzyme activity [59].

This experiment proved the MDCKII-bABCG2 cell line contained a functional AhR signaling pathway with inducible AhR target genes (AhRR, CYP1A1 and CYP1B1 mRNAs) and CYP1A enzyme activity by the known AhR ligand TCDD.

### Identification of AhR-activating pesticides

Several pesticides activate transcription factors, leading to increased expression and activity of their target genes [32]. The mRNA levels of AhR, AhRR, CYP1A1 and CYP1B1 were measured by qPCR while CYP1A enzyme activity was measured by EROD assay [14,15].

**Fig 1:**
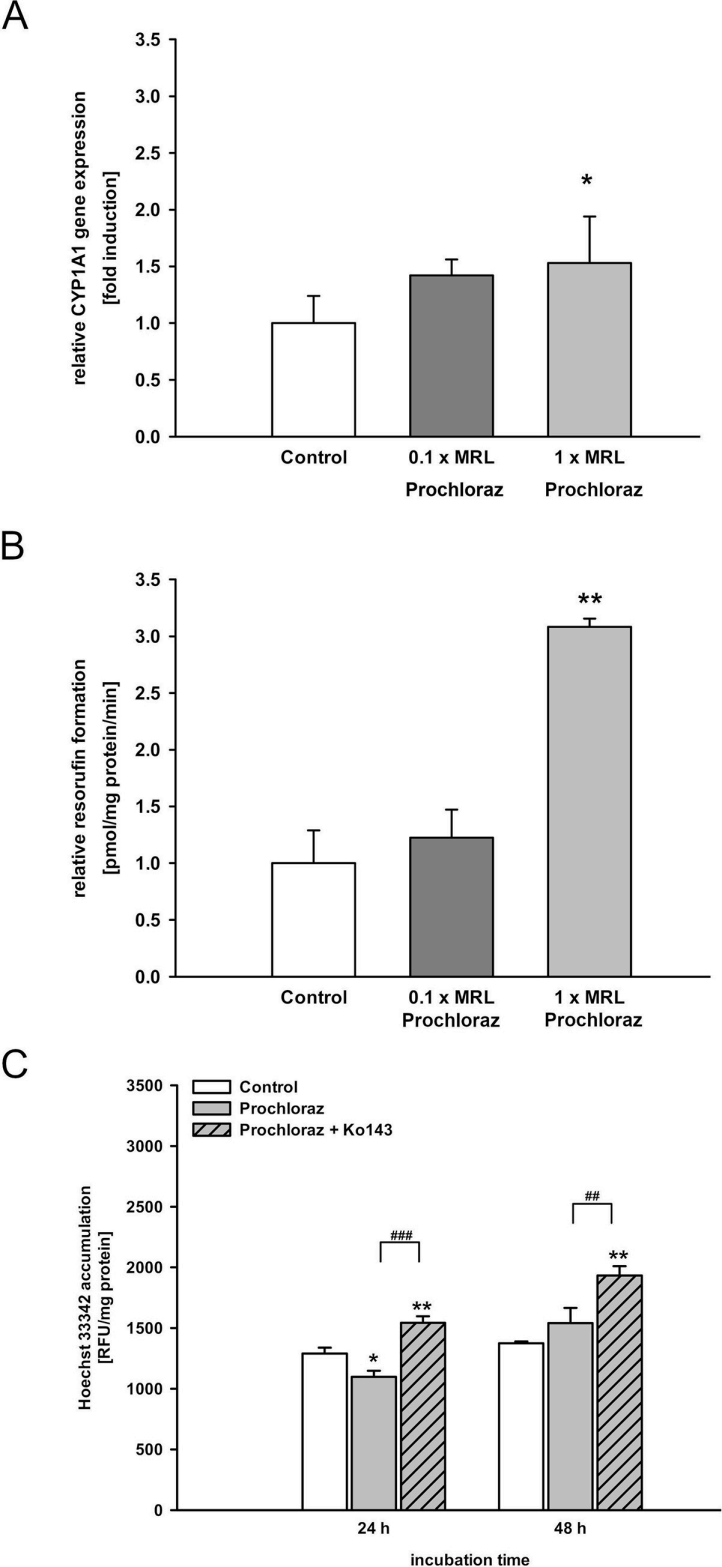
Impact of the positive control prochloraz on bABCG2 efflux activity, CYP1A1 gene expression and activity. **A./B.** MDCKII-bABCG2 cells were treated with prochloraz in 0.1- and 1-fold MRL concentration (8 nM, 80 nM) for 72 h. CYP1A1 mRNA expression and CYP1A activity were measured by qPCR and EROD assay. Data were normalized to control levels set as 1. **C.** The cells were treated with 1-fold MRL concentration of prochloraz (80 nM) or vehicle control (0.1% ethanol) for 24 h or 48 h. The intracellular Hoechst 33342 accumulation was measured in presence or absence of the ABCG2-inhibitor Ko143 (5 μM). Data are expressed as mean ± SEM (three independent experiments, one-way ANOVA with Tukey’s post hoc test or Fisher LSD post hoc test, * significant difference in comparison to the control: *** p ≤ 0.001, ** p ≤ 0.01, * p ≤ 0.05; # significantly different to Ko143: ### p ≤ 0.001, ## p ≤ 0.01, # p ≤ 0.05).

**Fig 2:**
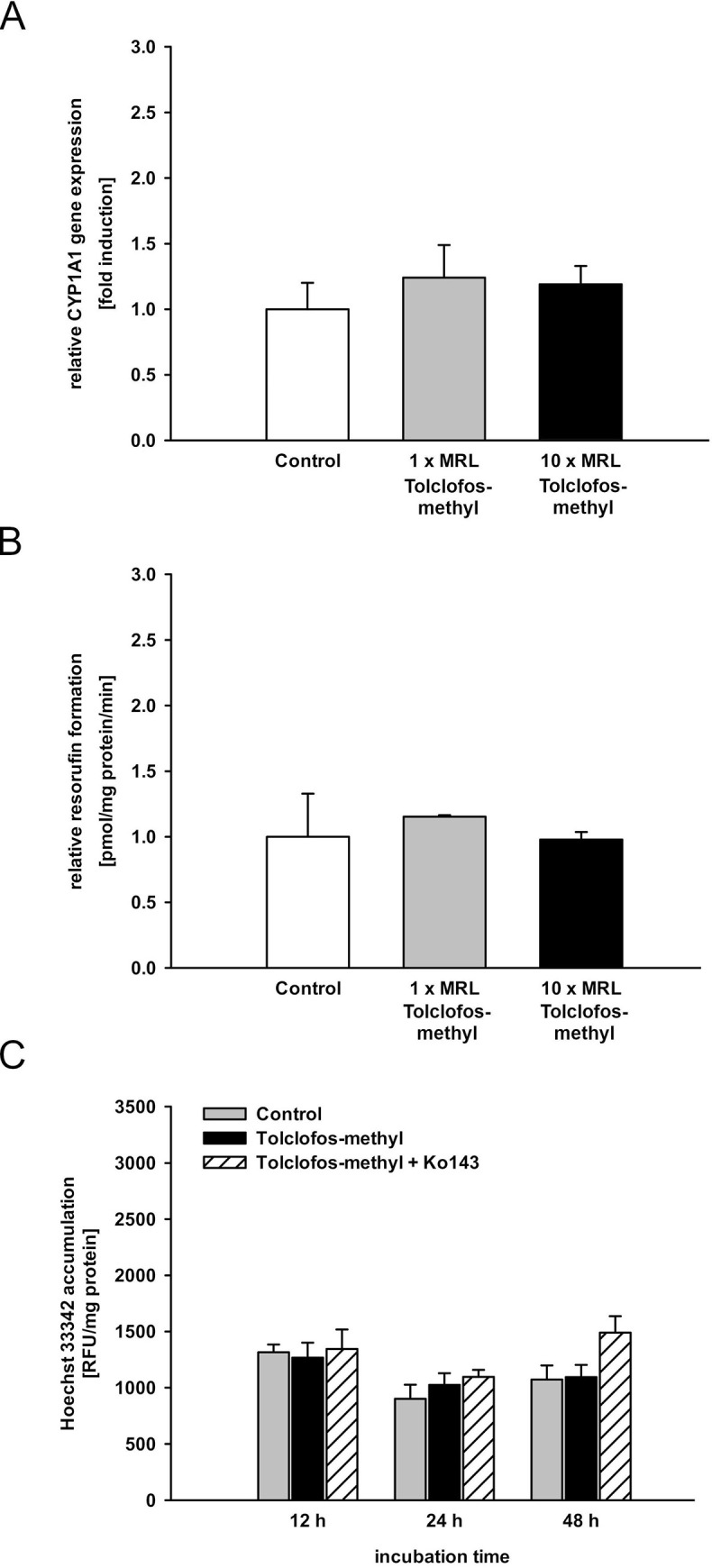
Impact of the negative control Tolclofos-methyl on bABCG2 efflux activity, CYP1A1 gene expression and activity. **A./B.** MDCKII-bABCG2 cells were treated with tolclofos-methyl in 1- and 10-fold MRL concentration (33 nM, 330 nM) for 72 h. CYP1A1 mRNA expression and CYP1A activity were measured by qPCR and EROD assay. Data were normalized to control levels set as 1. **C.** The cells were treated for 24 h or 48 h with 10-fold MRL concentration of tolclofos-methyl (330 nM) or vehicle control (0.1% methanol). The intracellular Hoechst 33342 accumulation was measured in presence or absence of the ABCG2-inhibitor Ko143 (5 μM). Data are expressed as mean ± SEM (three independent experiments, one-way ANOVA with Tukey’s post hoc test or Fisher LSD post hoc test, * significant difference in comparison to the control: *** p ≤ 0.001, ** p ≤ 0.01, * p ≤ 0.05; # significantly difference to Ko143: ### p ≤ 0.001, ## p ≤ 0.01, # p ≤ 0.05).

The positive control prochloraz (1-fold MRL) significantly increased CYP1A1 mRNA expression (1.5-fold, Fig 1A) and CYP1A activity (3-fold, Fig 1B), while CYP1B1, AhR, AhRR or ARNT expression levels were not altered. In accordance with our previous studies [28,29,51], neither the negative control tolclofos-methyl nor the solvents had an impact on the AhR target genes CYP1A1 (Fig 2A and 2B, S5 Fig), CYP1B1, or AhRR (S3 Table, S5 Fig).

**Fig 3:**
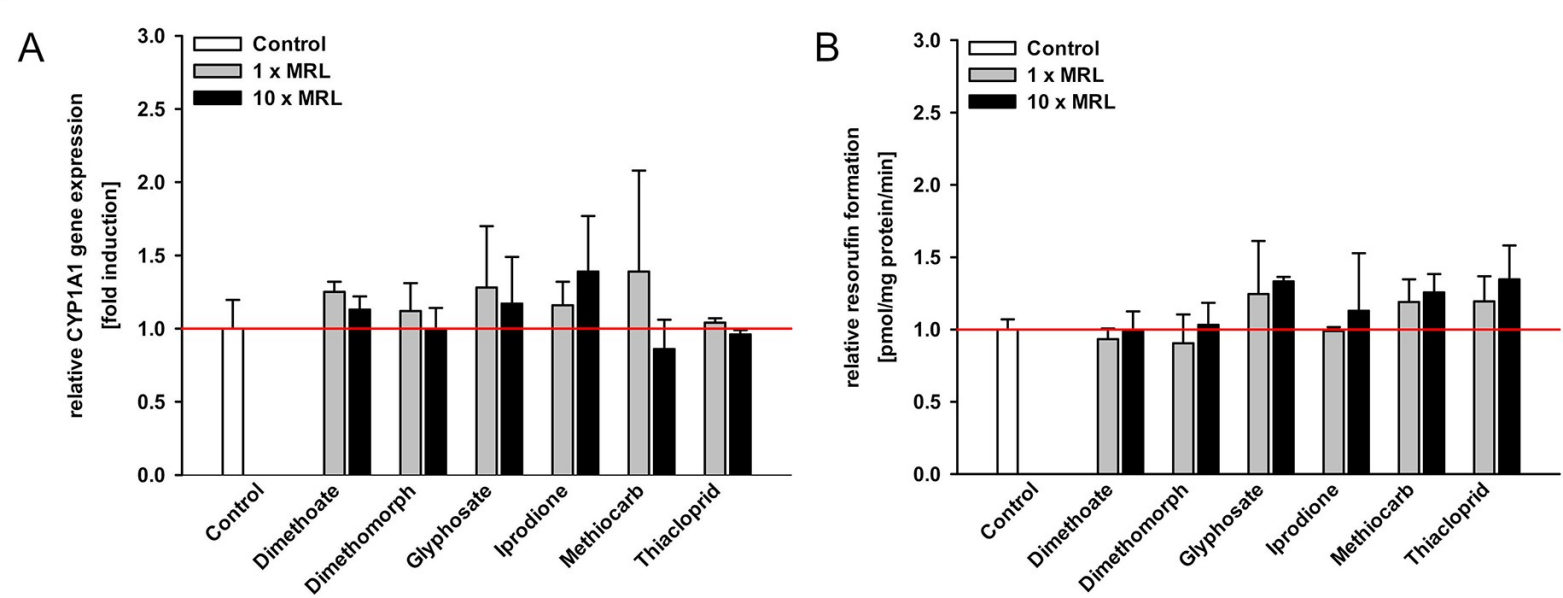
Impact of non AhR-activating pesticides on CYP1A1 gene expression and CYP1A activity in MDCKII-bABCG2 cells. The cells were treated with selected pesticides in 1- and 10-fold MRL concentration (Table 1) for 72 h. **A.** CYP1A1 mRNA expression was examined by qPCR and **B.** CYP1A activity was detected by EROD assay. All presented data were normalized to control levels set as 1 and expressed as mean ± SEM (three independent experiments, one-way ANOVA with Tukey’s post hoc test or Fisher LSD post hoc test, * significant difference in comparison to the control: * p ≤ 0.05).

The pesticides dimethoate, dimethomorph, glyphosate, iprodione, methiocarb, and thiacloprid did not alter CYP1A1 mRNA expression or CYP1A-mediated EROD activity (Fig 3A and 3B) in a result comparable to the negative control tolclofos-methyl (Fig 2A and 2B). These five pesticides were therefore classified as “non-AhR-activating pesticides” in accordance with the literature [8,9].

**Fig 4:**
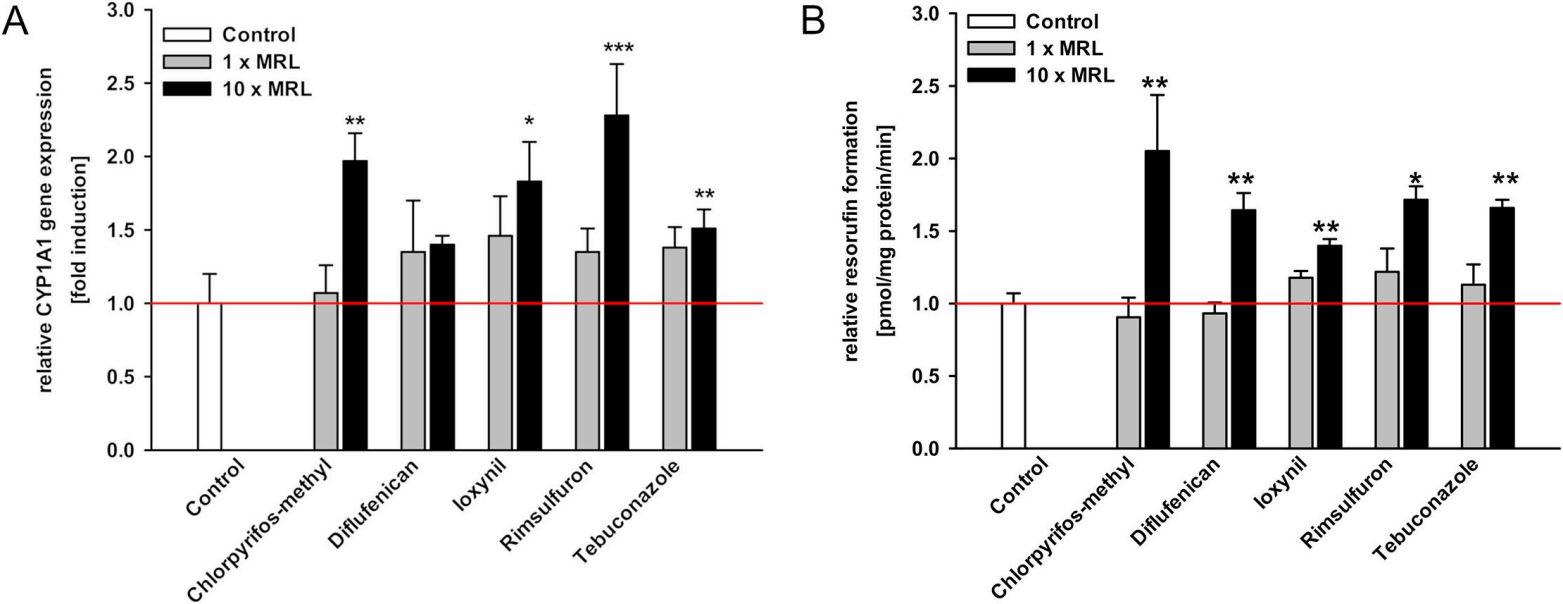
Increased CYP1A1 mRNA expression and CYP1A activity after treatment of MDCKII-bABCG2 cells with AhR-activating pesticides. MDCKII-bABCG2 cells were treated with selected pesticides in 1- and 10-fold MRL concentration (Table 1) for 72 h. A. Gene expression analysis of CYP1A1 and B. EROD assay were performed. All data were normalized to control levels set as 1 and expressed as mean ± SEM (three independent experiments, one-way ANOVA with Tukey’s post hoc test or Fisher LSD post hoc test, * significant difference in comparison to the control: *** p ≤ 0.001, ** p ≤ 0.01, * p ≤ 0.05).

As shown in Fig 4A, chlorpyrifos-methyl, ioxynil, rimsulfuron and tebuconazole significantly increased CYP1A1 mRNA levels by at least 50% compared to control levels. 10-fold MRL concentrations of these pesticides were also significantly increase EROD activity (Fig 4B). In contrast to TCDD (S3B and S3C Fig), none of these pesticides altered AhRR or CYP1B1 mRNA expression (S3 Table). CYP1A1 has previously been identified as the most inducible AhR target gene [57] which could explain why the tested pesticides only impact CYP1A1. Furthermore, a TCDD-mediated AhR induction is up to 500.000-fold stronger than prochloraz [9], potentially amplifying its impact to the other AhR target genes.

While treatment with 10-fold MRL of diflufenican only slightly increased CYP1A1 mRNA expression, EROD activity was doubled (Fig 4B). Therefore, chlorpyrifos-methyl, diflufenican, ioxynil, rimsulfuron, and tebuconazole were classified as “AhR-activating pesticides”.

### AhR-activating pesticides influence the bABCG2 efflux activity

The Hoechst 33342 accumulation assay was used to assess bABCG2-inducing pesticides. To rule out the direct interaction of applied pesticides with the bABCG2 transporter, cells were washed twice with PBS following pesticide exposure. Incubation with Hoechst 33342 then followed in the absence of pesticide.

**Fig 5:**
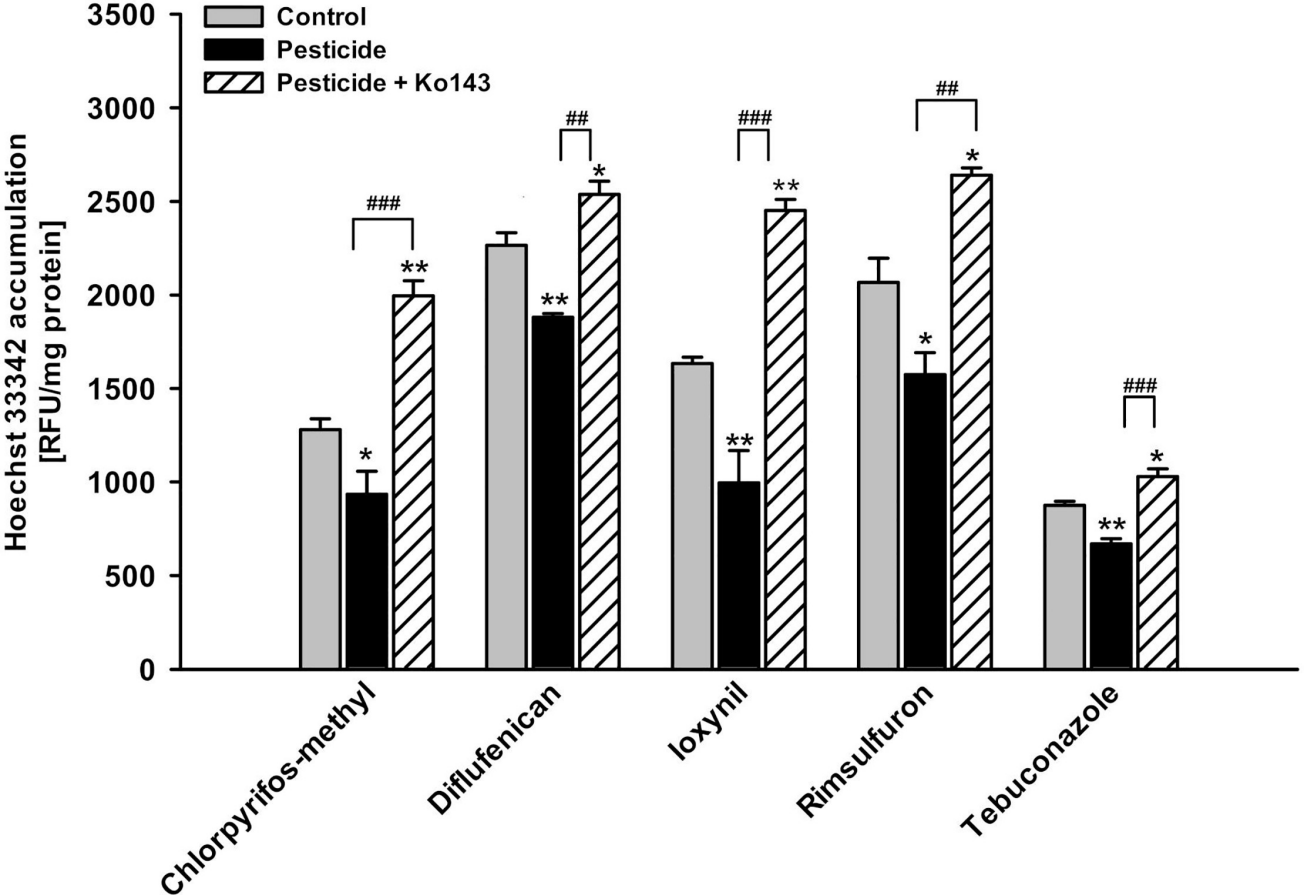
Impact of AhR-activating pesticides on the bABCG2-mediated Hoechst 33342 accumulation after 24 h incubation. MDCKII-bABCG2 cells were treated with the selected pesticides in 10-fold MRL concentration (Table 1) for 24 h and the Hoechst 33342 accumulation assay was performed in presence or absence of the ABCG2-inhibitor Ko143 (5 μM). Data are expressed as mean ± SEM (three independent experiments, one-way ANOVA with Fisher LSD post hoc test, * significant difference in comparison to the control: *** p ≤ 0.001, ** p ≤ 0.01, * p ≤ 0.05; # significantly different to Ko143: ### p ≤ 0.001, ## p ≤ 0.01, # p ≤ 0.05).

MDCKII-bABCG2 cells contain 98 base pairs of the 5'-UTR including DREs in front of the bABCG2 gene [22]. After ligand-binding, the AhR-ligand-ARNT-complex binds to the DREs, inducing bABCG2 gene transcription and subsequent secretion of bABCG2 substrates [17,18,29]. In line with previous experiments [29,22,51], treatment of the cells with the ABCG2 inhibitor Ko143 (5 μM) significantly increased the intracellular Hoechst 33342 amount in comparison to solvent- and pesticide-treated cells (Figs 1C, 2C and 5C). This proved that the bABCG2 transporter mediates Hoechst secretion. Elevated ABCG2 efflux activity is therefore represented by observing a decreased intracellular accumulation of the ABCG2 substrate Hoechst 33342.

Cells treated with 1-fold MRL of the positive control prochloraz for 24 h significantly decreased Hoechst 33342 levels by approximately 20% (Fig 1C). The same results were observed after 48 h of exposure (Fig 1C). The negative control tolclofos-methyl (10-fold MRL) had no impact on the bABCG2 efflux activity (Fig 2C).

Cells incubated with 10-fold MRL of all previously identified AhR-activating pesticides significantly decrease the level of intracellular Hoechst after 24 h when compared to their respective solvent controls (Fig 5 and S6 Fig). All identified AhR-activating pesticides, chlorpyrifos-methyl, diflufenican, rimsulfuron, ioxynil and tebuconazole (10-fold MRL) were therefore proven to induce the bABCG2 efflux activity.

As shown in Fig 5 and S6 Fig, this result was only detectable after the cells were incubated with pesticide for 24 h, indicating that treatment with AhR-activating pesticides for 24 h represents the best experimental set-up to detect altered transporter activity in this approach.

## Conclusion

The current pesticide approval process relies on animal experiments conducted with lactating goats or cows. To reduce the need for these experiments and implement the 3R principle in practice, the MDCKII-bABCG2 cell model was investigated as an *in vitro* tool to identify AhR-activating and bABCG2-inducing pesticides.

MDCKII-bABCG2 cells contain an inducible AhR pathway where AhR activation increases the expression of typical AhR target genes, particularly CYP1A1. Thus, the EROD assay represents appropriate tool to detect AhR-activating pesticides.

Similar to the positive control prochloraz, 10-fold MRL concentrations of chlorpyrifos-methyl, diflufenican, ioxynil, rimsulfuron and tebuconazole activated the AhR pathway and increased bABCG2 efflux activity. As concluded by Chedik et al. 2018 [32], an increased efflux activity eliminates substrates faster from the body. In the bovine mammary gland, the enhanced secretion of bABCG2-substrates into milk, such as drugs and toxins, creates a potential risk to consumers.

Overall, the presented approach is an appropriate *in vitro* tool to reduce the number of animals required for residue depletion studies.

## Supporting information

S1 Raw data (XLSX)

S1 Table qPCR assay parameters: Efficiency, linearity and dynamic range. (PDF)

S2 Table Internal Control Genes (ICGs) used for the relative quantification analysis of data. (PDF)

S3 Table CYP1B1, AhR, AhRR and ARNT mRNA expression in untreated and treated MDCKII-bABCG2 cells. Data were normalized to control levels and are expressed as fold change of relative quantification value (RQ) in arbitrary units (AU) (mean ± SEM, N = 3, n = 6, one-way ANOVA with Tukey’s post hoc test, significant differences are shaded in grey, ^a^ significantly different to the control, ^b^ significant difference between 1- and 10-fold MRL concentration, ^aaa, bbb^ p ≤ 0.001; ^aa, bb, cc^ p ≤ 0.01; ^a, b^ p ≤ 0.05).
(PDF)

S1 Fig Cytotoxicity of prochloraz, tolclofos-methyl, ioxnyil, chlorpyrifos-methyl, diflufenican, dimethoate and dimethomorph. MDCKII cells were incubated with pesticides in increasing concentrations for 72 h. Cell viability was measured by water soluble tetrazolium-1 (WST-1) assay. Data were normalized to control levels and are expressed as percentage of cell viability (mean ± SEM, N = 2, n = 12, one-way ANOVA with Holm-Šidák post hoc test, * significant difference in comparison to the control: *** p ≤ 0.001, ** p ≤ 0.01, * p ≤ 0.05).
(PDF)

S2 Fig Cytotoxicity of glyphosate, thiacloprid, iprodione, methiocarb, rimsulfuron and tebuconazole. MDCKII cells were incubated with pesticides in increasing concentrations for 72 h. Cell viability was measured by water soluble tetrazolium-1 (WST-1) assay. Data were normalized to control levels and are expressed as percentage of cell viability (mean ± SEM, N = 2, n = 12, one-way ANOVA with Holm-Šidák post hoc test, * significant difference in comparison to the control: *** p ≤ 0.001, ** p ≤ 0.01, * p ≤ 0.05).
(PDF)

S3 Fig Effects of AhR-inducing TCDD upon gene expression of the AhR gene battery. MDCKII cells were incubated with TCDD (1 nM, 10 nM) for 72 h followed by gene expression analysis on CYP1A1 (A), CYP1B1 (B), AhRR (C) and AhR (D). Data were normalized to control levels and are expressed as fold change of relative quantification value (RQ) in arbitrary units (AU) (mean ± SEM, N = 3, n = 6, one-way ANOVA with Tukey’s post hoc test, *** indicate significant differences in comparison to the control with p ≤ 0.001).
(PDF)

S4 Fig Effects of PCB101 upon gene expression of the AhR gene battery. MDCKII cells were incubated with PCB101 (10 nM, 100 nM) for 72 h followed by gene expression analysis on CYP1A1 (A), CYP1B1 (B), AhRR (C) and AhR (D). Data were normalized to control levels and are expressed as fold change of relative quantification value (RQ) in arbitrary units (AU) (mean ± SEM, N = 3, n = 6, one-way ANOVA with Tukey’s post hoc test, * significant differences in comparison to the control, *** p ≤ 0.001; ** p ≤ 0.01; * p ≤ 0.05).
(PDF)

S5 Fig Effects of the solvents used to dissolve the pesticides and dioxins upon gene expression of CYP1A1 (A), CYP1B1 (B), AhRR (C) and AhR (D). MDCKII-bABCG2 cells were incubated with solvents, listed in Table **1**, for 72 h. Gene expression analysis was subsequently carried out on CYP1A1 (A), CYP1B1 (B), AhRR (C) and AhR (D). Data were normalized to control levels and are expressed as fold change of relative quantification value (RQ) in arbitrary units (AU) (mean ± SEM, N = 3, n = 6, one-way ANOVA with Tukey’s post hoc test, level of significance p ≤ 0.05).
(PDF)

S6 Fig Hoechst 33342 accumulation in MDCKII cells after 48 h incubation with AhR-activating pesticides. MDCKII-bABCG2 cells were treated with the selected pesticides in 10-fold MRL concentration (Table 1) for 48 h and the Hoechst 33342 accumulation assay was performed in presence or absence of the ABCG2-inhibitor Ko143 (5 μM). Data are expressed as mean ± SEM (three independent experiments, one-way ANOVA with Fisher LSD post hoc test, * significant difference in comparison to the control: *** p ≤ 0.001, ** p ≤ 0.01, * p ≤ 0.05; # significantly different to Ko143: ### p ≤ 0.001, ## p ≤ 0.01, # p ≤ 0.05).
(PDF)

## Abbreviations

ABCG2: ATP-binding cassette subfamily G 2
AhR: aryl hydrocarbon receptor
AhRR: aryl hydrocarbon receptor repressor
ARNT: aryl hydrocarbon receptor nuclear translocator
ATP5: mitochondrial ATP synthase 5
AU: arbitrary units
bABCG2: bovine ABCG2
BCA: bicinchoninic acid assay
BCRP: breast cancer resistance protein
bHLH/PAS: basic-region helix-loop-helix/period aryl hydrocarbon receptor nuclear translocator-single minded
CCZ1: vacuolar protein trafficking and biogenesis associated homologue
C_P_: crossing point or cycle number at detection threshold
CYP1A1: cytochrome P450 family 1, subfamily A, isoform 1
CYP1B1: cytochrome P450 family 1, subfamily B, isoform 1
DRE: dioxin response elements
EROD: 7-ethoxyresorufin-*Ο*-deethylase
HPRT1: hypoxanthine phosphoribosyl transferase 1
HSP90: heat shock protein 90
ICG: internal control gene
IC_50_: inhibitory concentration
MDCKII: Madin-Darby canine kidney II cells
MRL: maximum residue level
N: number of independent experiments
n: number of technical replicates
n.a.: not available
PBMEC: primary bovine mammary epithelial cells
PCB101: 2,2’,4,5,5’-pentachlorobiphenyl
qPCR: quantitative real time polymerase chain reaction
RFU: relative fluorescence unit
RPL32: ribosomal protein L32
RPL8: ribosomal protein L8
RPS5: ribosomal protein S5
RQ: relative quantification value
SEM: standard error of the mean
TCDD: 2,3,7,8-tetrachlorodibenzo-*p*-dioxin
UPL: universal probe library
UTR: untranslated region
WST-1: water-soluble tetrazolium 1
XAP2: hepatitis B virus protein X-associated protein 2
