# Supplementary material for: AhR-activating pesticides increase the bovine ABCG2 efflux activity in MDCKII-bABCG2 cells
Source: PLoS One. 2020 Aug 7;15(8):e0237163. doi: 10.1371/journal.pone.0237163 (PMC7413513; doi:10.1371/journal.pone.0237163)
Supplement: S1 Table — (PDF) [file pone.0237163.s002.pdf]

S1 Table. qPCR assay parameters: efficiency, linearity and dynamic range.

Gene	Efficiency (%)	Error	Dynamic range
AHR	93.9	0.008	24.51 – 33.98
AHRR	99.4	0.034	33.12 – 37.67
ARNT	95.9	0.016	25.43 – 35.55
CYP1A1	94.4	0.024	30.17 – 37.07
CYP1B1	90.4	0.004	25.56 – 32.59
ATP5B	99.5	0.006	17.90 – 28.89
CCZ1	101.6	0.008	22.59 – 33.36
HPRT1	97.7	0.004	21.27 – 30.99
RPL8	95.9	0.007	18.56 – 29.89
RPL32	98.5	0.019	23.01 – 32.34
RPS5	98.2	0.005	19.93 – 31.01
