# Supplementary material for: AhR-activating pesticides increase the bovine ABCG2 efflux activity in MDCKII-bABCG2 cells
Source: PLoS One. 2020 Aug 7;15(8):e0237163. doi: 10.1371/journal.pone.0237163 (PMC7413513; doi:10.1371/journal.pone.0237163)
Supplement: S2 Table — (PDF) [file pone.0237163.s003.pdf]

S2 Table. Internal Control Genes (ICGs) used for the relative quantification analysis of data.

Compound used for cell treatment	ICGs used for normalization
TCDD	ATP5B, CCZ1, HPRT1, RPL8, RPL32, RPS5
PCB101	ATP5B, CCZ1, HPRT1, RPL8, RPL32, RPS5
Prochloraz	ATP5B, CCZ1, HPRT1, RPL8, RPL32, RPS5
Tolchlofos-methyl	CCZ1, HPRT1, RPL8, RPS5
Chlorpyrifos-methyl	ATP5B, CCZ1, HPRT1, RPL8, RPL32
Diflufenican	ATP5B, CCZ1, HPRT1, RPL32, RPS5
Dimethoate	ATP5B, CCZ1, HPRT1, RPL32, RPS5
Dimethomorph	HPRT1, RPL8, RPL32, RPS5
Glyphosate	CCZ1, HPRT1, RPL8, RPL32, RPS5
Ioxynil	CCZ1, HPRT1, RPL8, RPL32, RPS5
Iprodione	ATP5B, CCZ1, HPRT1, RPL32, RPS5
Methiocarb	ATP5B, CCZ1, HPRT1, RPL8, RPL32, RPS5
Rimsulfuron	RPL32
Tebuconazole	ATP5B, CCZ1, HPRT1, RPL32
Thiacloprid	ATP5B, CCZ1, HPRT1, RPL8, RPL32, RPS5
