# Supplementary material for: AhR-activating pesticides increase the bovine ABCG2 efflux activity in MDCKII-bABCG2 cells
Source: PLoS One. 2020 Aug 7;15(8):e0237163. doi: 10.1371/journal.pone.0237163 (PMC7413513; doi:10.1371/journal.pone.0237163)
Supplement: S3 Table — Data were normalized to control levels and are expressed as fold change of relative quantification value (RQ) in arbitrary units (AU) (mean ± SEM, N = 3, n = 6, one-way ANOVA with Tukey’s post hoc test, significant differences are shaded in grey, a significantly different to the control, b significant difference between 1- and 10-fold MRL concentration, aaa, bbb p ≤ 0.001; aa, bb, cc p ≤ 0.01; a, b p ≤ 0.05). (PDF) [file pone.0237163.s004.pdf]

S3 Table. CYP1B1, AhR, AhRR and ARNT mRNA expression in untreated and treated MDCKII-bABCG2 cells.

Pesticide	Experimental groups	Fold change (AU) Mean ± SEM			
		CYP1B1	AhR	AhRR	ARNT
**Prochloraz**	control	1.00 ± 0.26	1.00 ± 0.08	1.00 ± 0.63	1.00 ± 0.22
	10 nM	1.54 ± 0.26	1.29 ± 0.16	0.93 ± 0.06	1.37 ± 0.14
	100 nM	1.25 ± 0.20	1.05 ± 0.20	0.74 ± 0.04	1.31 ± 0.05
**Tolchlofos-methyl**	control	1.00 ± 0.22	1.00 ± 0.19	1.00 ± 0.20	1.00 ± 0.19
	33 nM	0.83 ± 0.23	1.13 ± 0.09	0.77 ± 0.06	0.74 ± 0.09[a]
	330 nM	0.97 ± 0.32	1.11 ± 0.21	0.87 ± 0.16	0.82 ± 0.08
**Chlorpyrifos-methyl**	control	1.00 ± 0.21	1.00 ± 0.20	1.00 ± 0.20	1.00 ± 0.19
	37 nM	0.90 ± 0.12	0.85 ± 0.10	1.38 ± 0.19	1.18 ± 0.10
	370 nM	1.29 ± 0.08	0.87 ± 0.12	1.23 ± 0.04	1.22 ± 0.06
**Diflufenican**	control	1.00 ± 0.13	1.00 ± 0.09	1.00 ± 0.09	1.00 ± 0.04
	25 nM	0.72 ± 0.14	0.95 ± 0.11	1.20 ± 0.13	0.67 ± 0.08[aa]
	250 nM	0.89 ± 0.13	1.04 ± 0.16	1.08 ± 0.13	0.76 ± 0.04[a]
**Dimethoate**	control	1.00 ± 0.13	1.00 ± 0.09	1.00 ± 0.09	1.00 ± 0.04
	44 nM	0.69 ± 0.13	1.04 ± 0.15	0.66 ± 0.03	0.67 ± 0.03[a]
	440 nM	0.50 ± 0.05	0.87 ± 0.25	0.86 ± 0.11	0.60 ± 0.10[aa]
**Dimethomorph**	control	1.00 ± 0.13	1.00 ± 0.09	1.00 ± 0.09	1.00 ± 0.04
	52 nM	0.70 ± 0.13	0.98 ± 0.16	1.00 ± 0.06	1.04 ± 0.04
	520 nM	0.80 ± 0.16	1.24 ± 0.23	0.94 ± 0.10	1.10 ± 0.08
**Glyphosate**	control	1.00 ± 0.13	1.00 ± 0.09	1.00 ± 0.09	1.00 ± 0.04
	0.3 µM	0.72 ± 0.15	1.00 ± 0.15	0.89 ± 0.11	0.90 ± 0.11
	3.0 µM	0.85 ± 0.28	0.99 ± 0.18	0.89 ± 0.10	0.85 ± 0.10
**Iprodione**	control	1.00 ± 0.13	1.00 ± 0.09	1.00 ± 0.09	1.00 ± 0.04
	0.1 µM	0.70 ± 0.14	0.84 ± 0.17	0.77 ± 0.13	0.67 ± 0.10[a]
	1.0 µM	0.86 ± 0.19	0.89 ± 0.16	0.89 ± 0.14	0.75 ± 0.09
**Ioxynil**	control	1.00 ± 0.13	1.00 ± 0.09	1.00 ± 0.09	1.00 ± 0.04
	2.7 µM	0.86 ± 0.22	1.16 ± 0.06	0.75 ± 0.07	0.80 ± 0.08
	27 µM	0.79 ± 0.24	0.90 ± 0.06	0.86 ± 0.11	0.76 ± 0.13
**Methiocarb**	control	1.00 ± 0.12	1.00 ± 0.05	1.00 ± 0.10	1.00 ± 0.06
	44.4 nM	0.97 ± 0.36	0.91 ± 0.11	0.99 ± 0.29	1.02 ± 0.17
	444 nM	0.85 ± 0.31	0.83 ± 0.18	0.80 ± 0.17	0.90 ± 0.12
**Rimsulfuron**	control	1.00 ± 0.08	1.00 ± 0.04	1.00 ± 0.13	1.00 ± 0.04
	116 nM	0.71 ± 0.11	0.60 ± 0.03[aa]	0.83 ± 0.06	0.73 ± 0.09[a,b]
	1.16 µM	1.10 ± 0.15	0.77 ± 0.12[a]	1.03 ± 0.07	1.04 ± 0.10[b]
**Tebuconazole**	control	1.00 ± 0.08	1.00 ± 0.02	1.00 ± 0.13	1.00 ± 0.03
	324.9 nM	1.07 ± 0.07	0.91 ± 0.08	1.48 ± 0.39	0.81 ± 0.05[aaa]
	3.249 µM	0.99 ± 0.15	0.85 ± 0.07	0.97 ± 0.14	0.75 ± 0.03[bbb]
**Thiacloprid**	control	1.00 ± 0.08	1.00 ± 0.04	1.00 ± 0.13	1.00 ± 0.03
	197.9 nM	0.61 ± 0.01[a]	0.82 ± 0.08	0.63 ± 0.06	0.67 ± 0.04[aaa]
	1.979 µM	0.61 ± 0.08[a]	0.77 ± 0.04[a]	0.55 ± 0.06	0.60 ± 0.04[bbb]

Data were normalized to control levels and are expressed as fold change of relative quantification value (RQ) in arbitrary units (AU) (mean ± SEM, N = 3, n = 6, one-way ANOVA with Tukey's post hoc test, significant differences are shaded in grey, [a] significantly different to the control, [b] significant difference between 1- and 10-fold MRL concentration, [aaa, bbb] $p \leq 0.001$; [aa, bb, cc] $p \leq 0.01$; [a, b] $p \leq 0.05$).
