# Supplementary material for: AhR-activating pesticides increase the bovine ABCG2 efflux activity in MDCKII-bABCG2 cells
Source: PLoS One. 2020 Aug 7;15(8):e0237163. doi: 10.1371/journal.pone.0237163 (PMC7413513; doi:10.1371/journal.pone.0237163)
Supplement: S1 Fig — MDCKII cells were incubated with pesticides in increasing concentrations for 72 h. Cell viability was measured by water soluble tetrazolium-1 (WST-1) assay. Data were normalized to control levels and are expressed as percentage of cell viability (mean ± SEM, N = 2, n = 12, one-way ANOVA with Holm-Šidák post hoc test, * significant difference in comparison to the control: *** p ≤ 0.001, ** p ≤ 0.01, * p ≤ 0.05). (PDF) [file pone.0237163.s005.pdf]

S1 Fig. Cytotoxicity of prochloraz, tolclofos-methyl, ioxnyil, chlorpyrifos-methyl, diflufenican, dimethoate and dimethomorph.

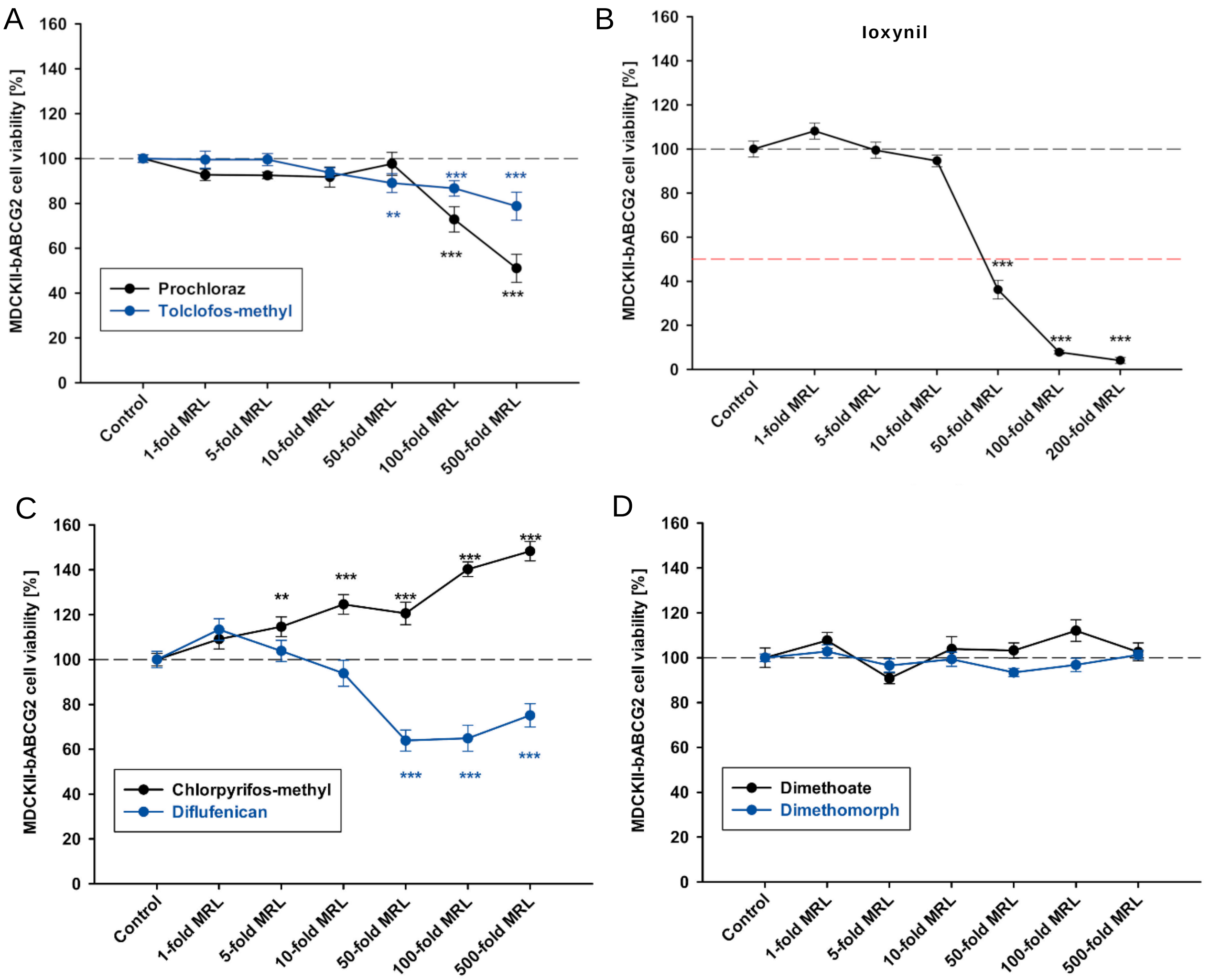

MDCKII cells were incubated with pesticides in increasing concentrations for 72 h. Cell viability was measured by water soluble tetrazolium-1 (WST-1) assay. Data were normalized to control levels and are expressed as percentage of cell viability (mean ± SEM, N = 2, n = 12, one-way ANOVA with Holm-Šidák post hoc test, * significant difference in comparison to the control: *** $p \leq 0.001$, ** $p \leq 0.01$, * $p \leq 0.05$).
