# Supplementary material for: AhR-activating pesticides increase the bovine ABCG2 efflux activity in MDCKII-bABCG2 cells
Source: PLoS One. 2020 Aug 7;15(8):e0237163. doi: 10.1371/journal.pone.0237163 (PMC7413513; doi:10.1371/journal.pone.0237163)
Supplement: S3 Fig — MDCKII cells were incubated with TCDD (1 nM, 10 nM) for 72 h followed by gene expression analysis on CYP1A1 (A), CYP1B1 (B), AhRR (C) and AhR (D). Data were normalized to control levels and are expressed as fold change of relative quantification value (RQ) in arbitrary units (AU) (mean ± SEM, N = 3, n = 6, one-way ANOVA with Tukey’s post hoc test, *** indicate significant differences in comparison to the control with p ≤ 0.001). (PDF) [file pone.0237163.s007.pdf]

S3 Fig. Effects of the solvents used to dissolve the pesticides and dioxins upon gene expression of CYP1A1 (A), CYP1B1 (B), AhRR (C) and AhR (D).

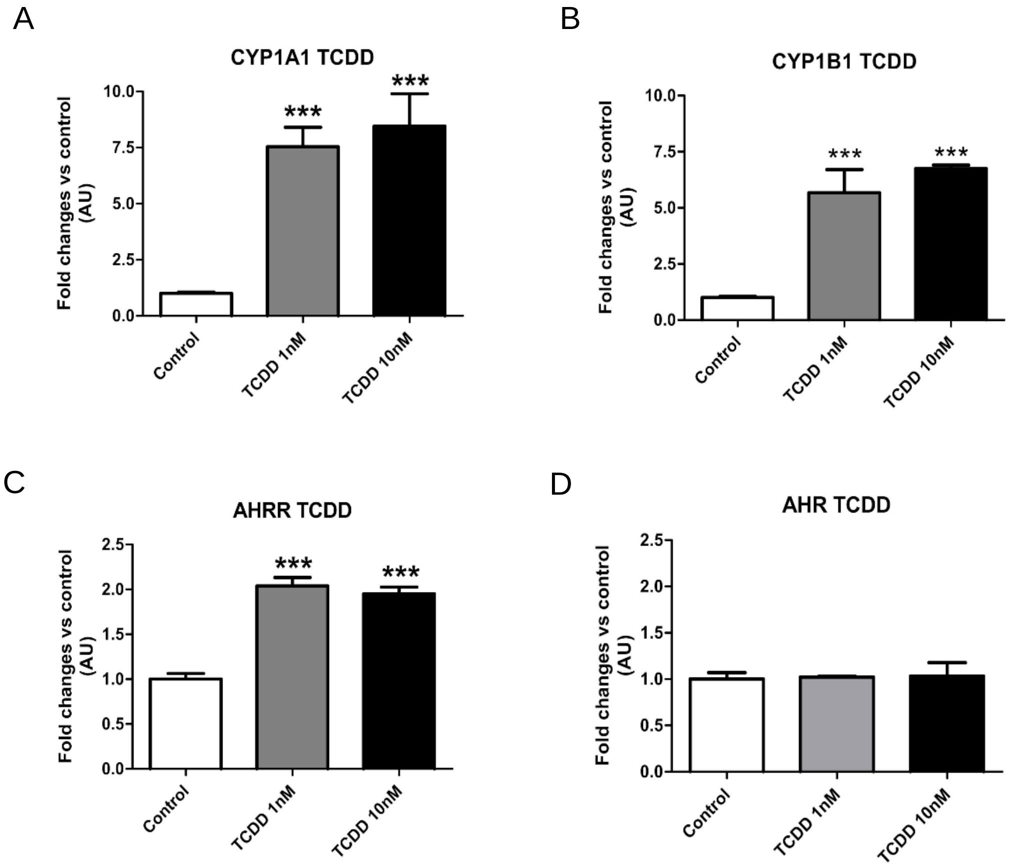

MDCKII cells were incubated with TCDD (1 nM, 10 nM) for 72 h followed by gene expression analysis on CYP1A1 (A), CYP1B1 (B), AhRR (C) and AhR (D). Data were normalized to control levels and are expressed as fold change of relative quantification value (RQ) in arbitrary units (AU) (mean ± SEM, N = 3, n = 6, one-way ANOVA with Tukey's post hoc test, *** indicate significant differences in comparison to the control with $p \leq 0.001$).
