# Supplementary material for: AhR-activating pesticides increase the bovine ABCG2 efflux activity in MDCKII-bABCG2 cells
Source: PLoS One. 2020 Aug 7;15(8):e0237163. doi: 10.1371/journal.pone.0237163 (PMC7413513; doi:10.1371/journal.pone.0237163)
Supplement: S4 Fig — MDCKII cells were incubated with PCB101 (10 nM, 100 nM) for 72 h followed by gene expression analysis on CYP1A1 (A), CYP1B1 (B), AhRR (C) and AhR (D). Data were normalized to control levels and are expressed as fold change of relative quantification value (RQ) in arbitrary units (AU) (mean ± SEM, N = 3, n = 6, one-way ANOVA with Tukey’s post hoc test, * significant differences in comparison to the control, *** p ≤ 0.001; ** p ≤ 0.01; * p ≤ 0.05). (PDF) [file pone.0237163.s008.pdf]

S4 Fig. Effects of PCB101 upon gene expression of the AhR gene battery.

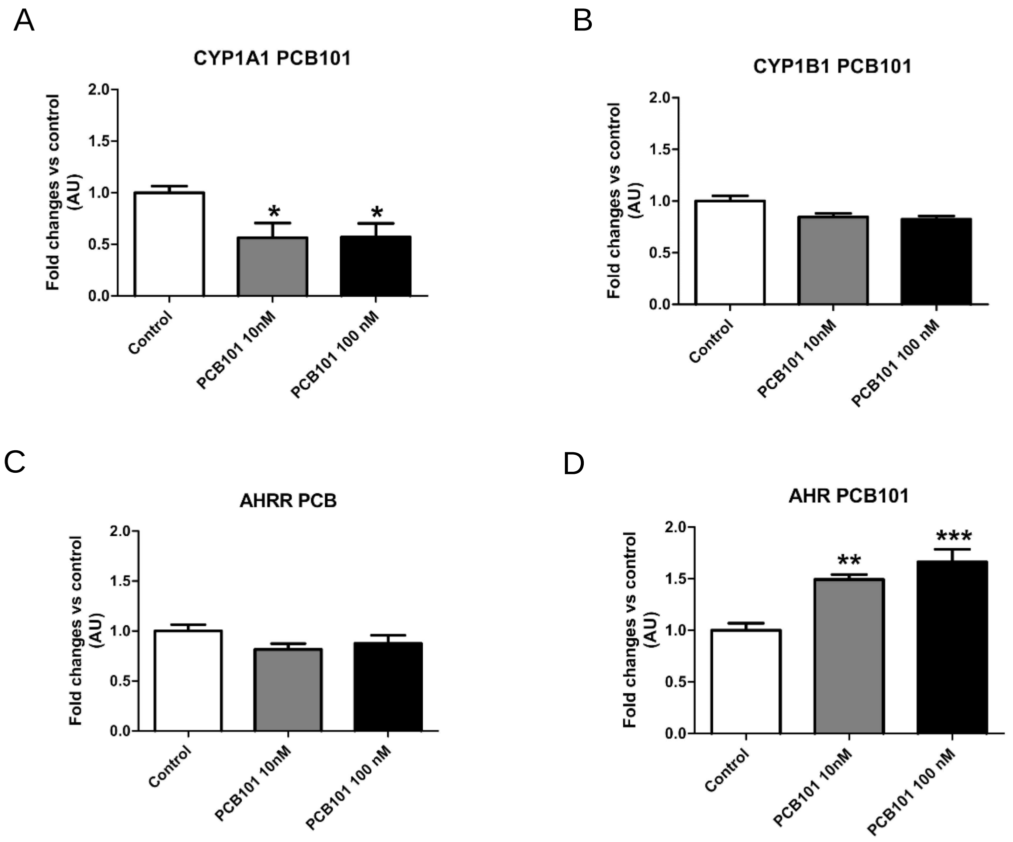

MDCKII cells were incubated with PCB101 (10 nM, 100 nM) for 72 h followed by gene expression analysis on CYP1A1 (A), CYP1B1 (B), AhRR (C) and AhR (D). The data were normalized to control levels and are expressed as fold change of relative quantification value (RQ) in arbitrary units (AU) (mean ± SEM, N = 3, n = 6, one-way ANOVA with Tukey's post hoc test, * significant differences in comparison to the control, *** $p \leq 0.001$; ** $p \leq 0.01$; * $p \leq 0.05$).
