# Supplementary material for: AhR-activating pesticides increase the bovine ABCG2 efflux activity in MDCKII-bABCG2 cells
Source: PLoS One. 2020 Aug 7;15(8):e0237163. doi: 10.1371/journal.pone.0237163 (PMC7413513; doi:10.1371/journal.pone.0237163)
Supplement: S5 Fig — Effects of the solvents used to dissolve the pesticides and dioxins upon gene expression of CYP1A1 (A), CYP1B1 (B), AhRR (C) and AhR (D). MDCKII-bABCG2 cells were incubated with solvents, listed in Table 1, for 72 h. Gene expression analysis was subsequently carried out on CYP1A1 (A), CYP1B1 (B), AhRR (C) and AhR (D). Data were normalized to control levels and are expressed as fold change of relative quantification value (RQ) in arbitrary units (AU) (mean ± SEM, N = 3, n = 6, one-way ANOVA with Tukey’s post hoc test, level of significance p ≤ 0.05). (PDF) [file pone.0237163.s009.pdf]

S5 Fig. Effects of the solvents used to dissolve the pesticides and dioxins upon gene expression of CYP1A1 (A), CYP1B1 (B), AhRR (C) and AhR (D).

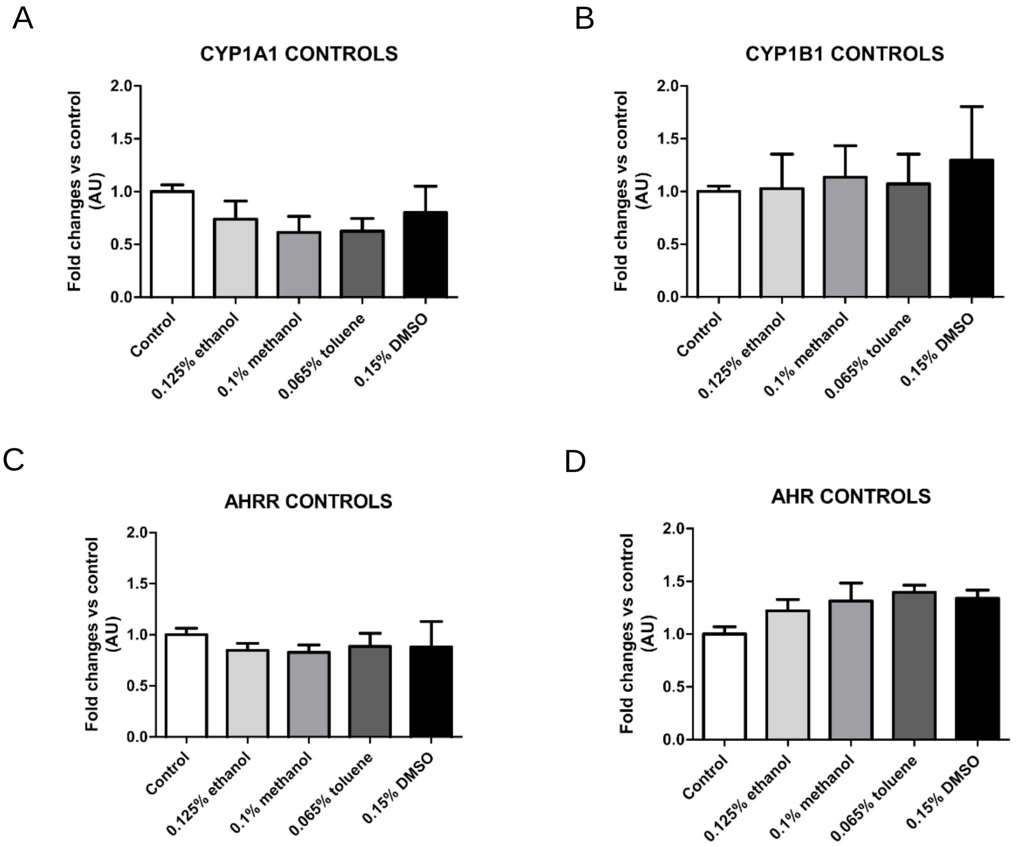

MDCKII-bABCG2 cells were incubated with solvents, listed in Table 1, for 72 h. Gene expression analysis was subsequently carried out on CYP1A1 (A), CYP1B1 (B), AhRR (C) and AhR (D). Data were normalized to control levels and are expressed as fold change of relative quantification value (RQ) in arbitrary units (AU) (mean ± SEM, N = 3, n = 6, one-way ANOVA with Tukey's post hoc test, level of significance $p \leq 0.05$).
