# Supplementary material for: AhR-activating pesticides increase the bovine ABCG2 efflux activity in MDCKII-bABCG2 cells
Source: PLoS One. 2020 Aug 7;15(8):e0237163. doi: 10.1371/journal.pone.0237163 (PMC7413513; doi:10.1371/journal.pone.0237163)
Supplement: S6 Fig — MDCKII-bABCG2 cells were treated with the selected pesticides in 10-fold MRL concentration (Table 1) for 48 h and the Hoechst 33342 accumulation assay was performed in presence or absence of the ABCG2-inhibitor Ko143 (5 μM). Data are expressed as mean ± SEM (three independent experiments, one-way ANOVA with Fisher LSD post hoc test, * significant difference in comparison to the control: *** p ≤ 0.001, ** p ≤ 0.01, * p ≤ 0.05; # significantly different to Ko143: ### p ≤ 0.001, ## p ≤ 0.01, # p ≤ 0.05). (PDF) [file pone.0237163.s010.pdf]

S6 Fig. Hoechst 33342 accumulation in MDCKII cells after 48 h incubation with AhR-activating pesticides.

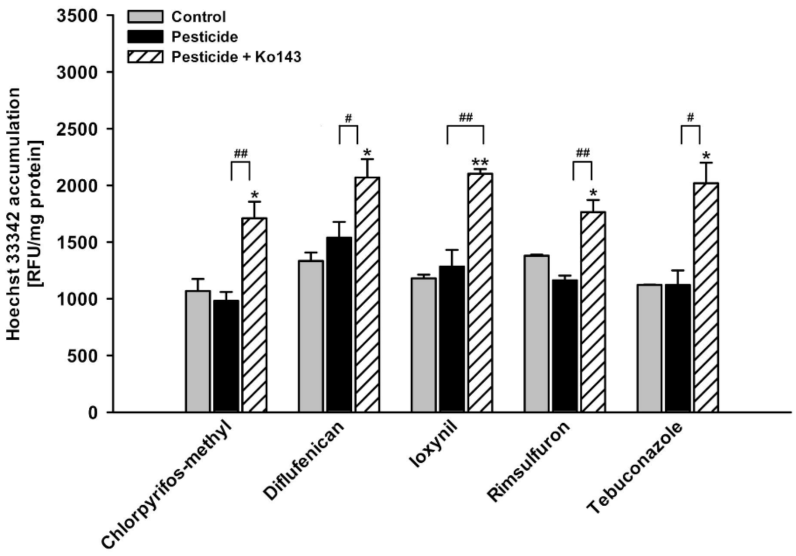

MDCKII-bABCG2 cells were treated with the selected pesticides in 10-fold MRL concentration (Table 1) for 48 h and the Hoechst 33342 accumulation assay was performed in presence or absence of the ABCG2-inhibitor Ko143 (5 µM). Data are expressed as mean ± SEM (three independent experiments, one-way ANOVA with Fisher LSD post hoc test, * significant difference in comparison to the control: *** $p \leq 0.001$, ** $p \leq 0.01$, * $p \leq 0.05$; # significantly different to Ko143: ### $p \leq 0.001$, ## $p \leq 0.01$, # $p \leq 0.05$).
